# A survey of the reformulation of Australian child-oriented food products

**DOI:** 10.1186/1471-2458-13-836

**Published:** 2013-09-11

**Authors:** Stephanie Savio, Kaye Mehta, Tuesday Udell, John Coveney

**Affiliations:** 1 Nutrition and Dietetics, School of Medicine, Flinders University, Sturt Road, Bedford Park, Adelaide, South Australia, Australia; 2 Heart Foundation, Hutt St, Adelaide, South Australia, Australia; 3 Flinders Prevention, Promotion and Primary Health Care, Flinders University, Sturt Road, Bedford Park, Adelaide, South Australia, Australia

**Keywords:** Reformulation, Children, Food industry, Policy, Food supply, Nutrition, Food, Child-oriented products

## Abstract

**Background:**

Childhood obesity is one of the most pressing public health challenges of the 21st century. Reformulating commonly eaten food products is a key emerging strategy to improve the food supply and help address rising rates of obesity and chronic disease. This study aimed to monitor reformulation of Australian child-oriented food products (products marketed specifically to children) from 2009–2011.

**Methods:**

In 2009, all child-oriented food products in a large supermarket in metropolitan Adelaide were identified. These baseline products were followed up in 2011 to identify products still available for sale. Nutrient content data were collected from Nutrient Information Panels in 2009 and 2011. Absolute and percentage change in nutrient content were calculated for energy, total fat, saturated fat, sugars, sodium and fibre. Data were descriptively analysed to examine reformulation in individual products, in key nutrients, within product categories and across all products. Two methods were used to assess the extent of reformulation; the first involved assessing percentage change in single nutrients over time, while the second involved a set of nutrient criteria to assess changes in overall healthiness of products over time.

**Results:**

Of 120 products, 40 remained unchanged in nutrient composition from 2009–2011 and 80 underwent change. The proportions of positively and negatively reformulated products were similar for most nutrients surveyed, with the exception of sodium. Eighteen products (15%) were simultaneously positively and negatively reformulated for different nutrients. Using percentage change in nutrient content to assess extent of reformulation, nearly half (n = 53) of all products were at least moderately reformulated and just over one third (n = 42) were substantially reformulated. The nutrient criteria method revealed 5 products (6%) that were positively reformulated and none that had undergone negative reformulation.

**Conclusion:**

Positive and negative reformulation was observed to a similar extent within the sample indicating little overall improvement in healthiness of the child-oriented food supply from 2009–2011. In the absence of agreed reformulation standards, the extent of reformulation was assessed against criteria developed specifically for this project. While arbitrary in nature, these criteria were based on reasonable assessment of the meaningfulness of reformulation and change in nutrient composition. As well as highlighting nutrient composition changes in a number of food products directed to children, this study emphasises the need to develop comprehensive, targeted and standardised reformulation benchmarks to assess the extent of reformulation occurring in the food supply.

## Background

### The childhood obesity problem

The World Health Organization (WHO) identifies childhood obesity as one of the most serious public health challenges of the 21st century [1]. In Australia, 20% of children aged 2–3 years were overweight or obese in 2007 [2] and one-quarter of children aged 5–17 years were overweight or obese in 2007-08 [3]. As overweight children are more likely to become overweight adults [4], preventing childhood overweight and obesity is a public health priority [5].

### Diet and the food supply

Poor diet plays an important role in the aetiology of childhood overweight and obesity [6] and the growing consumption of unhealthy convenience and snack foods [7] and high sugar foods and beverages [8,9] has been linked to increasing obesity rates, particularly in children. A global shift in diet and food supply towards increased intakes and accessibility of cheap, energy-dense foods and the persuasive marketing of these foods are identified as major contributing factors [10,11]. This trend has put a spotlight on the food supply and particularly the food industry and its putative role in the obesity problem [12,13]. Consequently, reformulation of commonly consumed food and beverage products to reduce nutrients associated with adverse health effects has been identified as a key emerging strategy in improving the food supply, thereby addressing chronic disease and achieving population nutrition goals [14-18].

In existing literature, reformulation is defined as a process that aims to improve the nutritional composition of a food or drink by removing nutrients associated with adverse health effects, decreasing portion size and, maintaining or increasing nutrients associated with positive health effects, while maintaining characteristics such as flavour, texture and shelf life [13].

### Reformulating child-oriented food products

Marketing foods to children is ‘big business’[19] and the majority of food products promoted to children, herein called ‘child-oriented food products’, are energy dense and nutrient poor [20]. This is particularly concerning as children develop long lasting food habits through repeated exposure to foods [21]. Furthermore, food preferences developed in childhood are likely to persist throughout a lifetime [22] and can, therefore, have both short-term and long-term consequences for health and weight status [8]. It is acknowledged that the prevention of overweight and obesity is unlikely to succeed if contributory environmental factors, including the food supply, are not addressed [15,23]. Reformulation of food and beverages marketed directly to children to improve nutritional profiles, may play a role in improving the child-oriented food supply. However, despite the obvious case for reformulating child-oriented food products, there are few publically promoted reformulation initiatives and little published research monitoring reformulation of child-oriented food products.

### Monitoring reformulation

In Australia, there are a number of initiatives promoting and monitoring reformulation of food products, although none specifically targeting the reformulation of child-oriented food products. For example, the National Heart Foundation of Australia promotes reformulation through their Tick program [24]; the Australian Division of the World Action on Salt & Health (AWASH) [25] sets targets for the maximum acceptable levels of sodium for major product categories; and the Food and Health Dialogue [26] has begun to collaborate with food industry to set voluntary reformulation targets for sodium and saturated fat for selected product categories. These initiatives also monitor industry progress towards these targets.

While food composition databases [27,28] and literature on food product composition [29,30] exist in Australia, there are few papers that report on nutrient *changes* within individual products over time and little published literature documenting reformulation of child-oriented food products. Additionally, the majority of literature monitoring reformulation over time focuses on single nutrients, instead of reformulation across multiple nutrients within a product’s nutrient profile. Much of this literature also focuses specifically on trans fatty acids (TFA) [31,32] and sodium [24,33,34], or documents the progress of individual companies [35,36] and, therefore, lacks perspective of how reformulation is affecting the overall food supply.

Monitoring reformulation may be hindered by ‘health by stealth’, an industry technique whereby product reformulation is not actively promoted in order to maintain consumer acceptance of reformulated products [37]. The health by stealth approach is particularly favoured in child-oriented food products due to the difficulty in persuading some children to eat overtly healthy foods [37,38]. This technique has facilitated consumer acceptance of product reformulations (eg. salt reductions [37]), however as a result, changes in product composition have not been promoted to the public or publically evaluated and thus much information relating to changes in such nutrient profiles is not widely accessible.

Given the lack of data concerning reformulation of child-oriented food products, the present study aimed to compare changes in the nutritional composition (ie. reformulation) of a set of child-oriented food products sold in Australia between 2009 and 2011. In doing so, it investigated the extent to which the nutrient compositions of Australian child-oriented food products have changed in this time period and whether these reformulations nutritionally improved or nutritionally worsened product compositions.

## Methods

### Product selection

A baseline survey was conducted in 2009 to identify all food and beverage products marketed to children for sale in a single large Woolworths supermarket in metropolitan Adelaide [39]. In Australia, two supermarket chains, Coles and Woolworths, currently control the majority of food sales [40]. Woolworths was selected as the representative supermarket for this study because it carries similar products to Coles, it has over 700 supermarkets nationwide and it services 13 million customers each week [41]. The Woolworths store chosen for the study (Westfield Marion), is one of the larger stores in metropolitan Adelaide. While Woolworths stores vary in size between metropolitan and rural settings, through personal communication with the Woolworths Marketing Manager (personal communication 30 September, 2009), the authors were assured that food and beverage product lines are very similar.

Child-oriented food products were identified using criteria adapted from previous studies [42-44]. Products were required to meet at least two of the following inclusion criteria:

• Words on the packaging referred to children, fun, play, physical activity or school

• Images on the packaging represented cartoons, popular personalities or celebrities, children, or pictures that appeal to children

• Packaging emphasised unusual shapes, unconventional flavours or bright colours

• Cross promotions and tie-ins with children’s television programs, merchandise, films or websites

• Premium offers (competitions, games, puzzles, toys and other giveaways) that appealed to children.

Exclusion criteria for products were as follows:

• Fast food products and food and beverage products sold outside of supermarkets

• Home brand products (products packaged and marketed under the brand of the retailer eg. Woolworths. Also known as private label products)

• Baby foods

• Seasonal products (eg. Christmas foods)

Multiple-sized packages of the same product were recorded as one item (eg. different sized cans of baked beans were recorded as one item) and products with only slight variations in nutrient content were recorded as one item (eg. one item was recorded to represent the 14 variations of Allen’s Party Mix lollies).

This baseline analysis, conducted in October 2009, yielded a sample of 157 products for sale in the supermarket, which met the above criteria of child-oriented [45]. Products in the baseline sample were matched to identical products for sale in August/September 2011 by visiting a range of supermarkets in metropolitan Adelaide, including the original and other Woolworths stores, Coles, Foodland and IGA stores, searching brand websites and direct telephone contact with manufacturing companies. Due to stock fluctuations in the Woolworths supermarket used in the 2009 baseline survey, a range of supermarkets and websites were visited in 2011 in order to re-identify as many of the baseline products as possible on follow-up. In total, 120 identical products were identified in 2011. A full list and description of products included in the sample is available from the corresponding author.

### Data collection

Product name, brand, company and Nutrition Information Panel (NIP) information were recorded for each product including nutrient content per serve and per 100 g. Data were entered into SPSS version 19. This paper presents results for six key nutrients collected: energy, total fat, saturated fat, sugars, sodium and fibre. These nutrients are identified as target nutrients for reformulation or are key nutrients in children’s diets [16,17].

### Data analysis

Products were grouped into product categories derived from the Healthy Kids Association Nutrient Criteria [46]. Devised by the Healthy Kids Association, this category specific nutrient criteria is used to classify food and beverage products for school canteens into Green ‘Fill the Menu’, Amber ‘Select Carefully’ and Red ‘Occasionally’ categories [46]. Products categories used in this study are based on the Healthy Kids criteria categories and are as follows: processed fruit, breakfast cereals, snack food bars, sweet biscuit, savoury dry snack foods, milk (incl. flavoured and unflavoured), cheese, yoghurt, dairy snack foods, ice cream, pasta/ noodles, and meat/ fish/ chicken/ alternatives. Other categories not found in the Healthy Kids criteria but used in this study are confectionary, jelly, and miscellaneous products.

Absolute and percentage change between 2009 and 2011 were calculated for each product for kilojoules, total fat, saturated fat, sugars, sodium and fibre content per 100 g. Data were subjected to descriptive analysis to assess the nature and extent of reformulation between 2009 and 2011 in individual products, key nutrients, product categories and across the sample as a whole.

#### Describing the direction of reformulation

While the term *reformulation* has previously been used to describe changes that increase the healthiness of foods [13], this paper uses the terms ‘positive reformulation’ or ‘positively reformulated’ to describe a change in a product’s nutrient profile to improve a product’s healthiness, i.e. a decrease in energy density, total fat, saturated fat, sugars or sodium content or an increase in fibre content. Conversely, the terms ‘negatively reformulated’ or ‘negative reformulation’ describe changes in nutrient composition that make a product less healthy, i.e. an increase in energy density, total fat, saturated fat, sugars or sodium content or a decrease in fibre content.

#### Products highly consumed by children

According to the 2007 National Children’s Diet and Physical Activity Survey [2], the product groups that contributed most to energy intake across age groups during the day prior to the survey were cereals, cereal products and cereal based products, and milk products and dishes. As changes in these products are likely to have a greater effect on the dietary intakes of Australian children, reformulation of products from these particular categories within the study sample was also subjected to descriptive analysis.

#### Assessing the magnitude of reformulation

There is no consensus in the literature on what level of change constitutes ideal or acceptable reformulation for any one nutrient. Food Standards Australia and New Zealand (FSANZ) defines “reduced energy/ fat/ saturated fat/ sugars/ sodium” as a change of at least 25% from the reference food [47], however this is not currently used to assess reformulation. The Australian Food and Health Dialogue [26] provides sodium and saturated fat reformulation targets for select product categories, but does not yet provide a comprehensive set of benchmarks for other nutrients and product groups. These ‘benchmarks’ would provide a gold standard to which the food industry could aim when reformulating their products.

Considering this lack of comprehensive standardised reformulation benchmarks, the present study used two methods to assess and classify the magnitude of reformulation observed in the sample.

#### Method one: percentage change method

The FSANZ definition [47] of ‘reduced’ ie. a 25% reduction in nutrient content (commonly total fat, saturated fat, sugar or sodium) is one of the only formalised standards to recognise positive changes to product nutrient compositions across all product categories in the Australian food supply. In the absence of agreed reformulation benchmarks, authors used this standard as a basis for categorising reformulation observed in this study into three levels of percentage change:

1. **0**–**9.99% change** (increase or decrease) in any nutrient: Negligible change, not reformulation

2. **10% - 24.99% change** (increase or decrease) in any nutrient: Moderate Reformulation

3. **≥25% change** (increase or decrease) in any nutrient: Substantial reformulation

Thus where reformulation is discussed collectively, it refers to any change ≥10%.

#### Method two: nutrient criteria method

Permission was granted by the Healthy Kids Association (HKA) [48] to use the Healthy Kids Association Nutrient Criteria [46] to assess the healthiness of products over time. The Healthy Kids Nutrient Criteria are based on the NSW Fresh Tastes @ School Healthy School Canteen Strategy [49] that advises that Green foods and drinks should be encouraged and promoted, Amber foods and drinks should not dominate the menu and large serve sizes should be avoided, and Red foods and drinks should not be sold on more than two occasions per term [46]. These criteria were chosen because they were specifically designed to assess the healthiness of Australian food products for children. Unlike other Australian school canteen guidelines that divide products into only two categories (ie. Red and Amber/Green), the Healthy Kids criteria have category specific nutrient criteria to classify products as either Green or Amber and products that fall outside these criteria are classified as Red by the Fresh Tastes @ School criteria. As a result, the Healthy Kids/Fresh Tastes @ School criteria divide products into three distinct groups. The benefit of this method is that it assesses foods, not just single nutrients.

Using this method, calcium content and percentage fruit content data were required to assess dairy products and processed fruit products, respectively, against the HKA nutrient criteria. As these data were not collected in the 2009 data set, dairy and processed fruit products were excluded from this analysis. Using the NIP data, 85 products from the sample were assessed against the HKA nutrient criteria and given a classification (red, amber or green) according to their nutrient profile in both 2009 and 2011. Any products that underwent a change in classification over time were deemed reformulated (eg. a change from Red to Amber or Amber to green or vice versa).

## Results

Of the 2009 baseline sample of 158, 120 identical products were identified in 2011 including 3 rebranded products. Between 2009 and 2011, nine products were discontinued and on follow-up in 2011, nutrition information could not be verified for 22 products, two products were deemed inappropriate and four were excluded due to inconsistencies in data collected. This paper reports on the reformulation of the 120 products for which nutrition information was collected in both 2009 and 2011.

A range of product categories were represented in the sample with confectionary products contributing nearly one third (28%, n = 34) of products surveyed. Forty (33%) products remained unchanged and 80 (67%) products underwent some positive or negative change to nutrient composition between 2009 and 2011.

### Reformulation of key nutrients

Table 1 shows that while a proportion of products were positively reformulated for each nutrient surveyed, in most cases, an almost equal proportion of products were negatively reformulated for the same nutrient. For example, total fat content was reduced in 11 (9%) products, but was increased in 12 (10%) products. Similarly, sugars were decreased in 13 (11%) products, but increased in 8 (7%). This occurred to a lesser degree with sodium reformulation, with 25 (21%) products positively reformulated for sodium and only 14 (12%) negatively reformulated.

**Table 1:** Positive and negative reformulation of key nutrients in a sample of Australian child-oriented food products of between 2009-2011

	**% of products in sample reformulated**					
	**Energy**	**Total fat**	**Saturated fat**	**Sugars**	**Sodium**	**Fibre**
**Positive reformulation**	5%	9%	16%	11%	21%	7%
**Negative reformulation**	3%	10%	10%	7%	12%	3%

Where positive reformulation is ≥10% decrease in energy, total fat, saturated fat, sugars or sodium content or ≥10% increase in fibre content and negative reformulation is ≥10% increase in energy, total fat, saturated fat, sugars or sodium content or ≥10% decrease in fibre content.

The most frequently positively reformulated nutrients were sodium, saturated fat and sugars. However, the most frequently negatively reformulated nutrients were also sodium, total and saturated fats and sugars (Table 1).

### Reformulation in products highly consumed by children

Cereals, cereal products and cereal based products, and milk products and dishes contributed most to children’s energy intake in Australia in 2007 [2]. Cereals, cereal products and cereal based products make up 12.5% (n = 15) of the current sample, while milk products and dishes make up 21% (n = 25) of the sample. Around half of cereals and cereal products surveyed (56%, n = 9) (breakfast cereals and pasta/noodles) and milk products and dishes surveyed (40%, n = 10) (milk, yoghurts, cheese, dairy snacks) were positively reformulated for at least one nutrient, with yoghurts the most positively reformulated out of all categories surveyed (88% n = 7). However, 31% (n = 5) of cereals and cereal products and 24% (n = 6) of milk products and dishes were negatively reformulated for at least one nutrient.

### Magnitude of reformulation

#### Method one: percentage change method

Of the 120 products surveyed, almost half (44%, n = 53) were classified as reformulated (≥10% change in at least one nutrient surveyed) (Table 2). Of these reformulated products, 42 (35% of sample) were substantially reformulated (≥25% change in at least one nutrient surveyed), whereas 11 products (9% of sample) underwent moderate reformulation (10% - 24.9% change in at least one nutrient surveyed with no substantial reformulation in any nutrient surveyed). Table 3 provides absolute and percentage change data for each of these 53 products for kilojoules, total fat, saturated fat, sugars, sodium and fibre.

**Table 2:** Levels of positive and negative reformulation in a sample child-oriented food products sold in Australia between 2009-2011

	**Reformulated products in sample**			
	**Total products**	**Products positively reformulated only**	**Products negatively reformulated only**	**Products both positively and negatively reformulated**
**No change in nutrient profile**	33% (n = 40)	-	-	-
**Negligible change** (<10% change)	23% (n = 27)	-	-	-
**Moderate reformulation** (10% - 24.9% change)	9% (n = 11)	3% (n = 4)	3% (n = 4)	15% (n = 18)
**Substantial reformulation** (≥25% change)	35% (n = 42)	15% (n = 18)	8% (n = 9)	

Positive reformulation is a decrease in energy, total fat, saturated fat, sugars or sodium content or an increase in fibre content. Negative reformulation is an increase in energy, total fat, saturated fat, sugars or sodium content or a decrease in fibre content.

Products that underwent negligible reformulation showed <10% change in energy, total fat, saturated fat, sugars, sodium or fibre content and showed no moderate or substantial reformulation between 2009 and 2011.

Moderate reformulation is a 10% - 24.9% change in energy, total fat, saturated fat, sugars, sodium or fibre content between 2009 and 2011 and not substantially reformulated for any of these nutrients.

Substantial reformulation is a change ≥25% in energy, total fat, saturated fat, sugars, sodium or fibre content between 2009 and 2011.

**Table 3:** Absolute and percentage change in nutrient composition in child-oriented products undergoing moderate or substantial reformulation between 2009 and 2011

**Product**	**Brand**	**Change kj**	**% Change kj**	**Change total fat (g)**	**% Change total fat**	**Change sat fat (g)**	**% Change sat fat**	**Change sugars (g)**	**% Change sugars**	**Change sodium (mg)**	**% Change sodium**	**Change fibre (g)**	**% Change fibre**
**Biscuits: sweet**													
Tiny Teddies hundreds and thousands	Arnotts	9	0.5	−0.6	−4.7	−1.1	−14.3	3.9	15.5	−93	−24.2	−0.3	−6.3
Uglies	Paradise	−20	−1.0	−0.8	−3.9	−2	−14.3	−1.2	−3.6	−20	−6.7	0.8	25.0
Wheelies	Old fashion foods	−205	−10.0	0	0.0	−2.3	−19.2	2	7.1	149	149.0		
Wagon wheels	Arnotts	−90	−4.7	−0.9	−5.4	−1.3	−10.4	0.9	2.1	−6	−5.0		
**Breakfast cereals**													
Cheerios	Uncle Tobys	0	0.0	0	0.0	−0.4	−40.0	0	0.0	−110	−22.0	0	0.0
Froot loops	Kelloggs	0	0.0	0	0.0	0	0.0	0	0.0	60	14.6	0	0.0
Oats So tasty-now oats smooth & tasty	Uncle Tobys	−10	−0.6	−0.4	−5.8	−0.4	−25.0	−1.9	−7.1	5	20.0	0.2	2.8
Honey wheats	Sanitarium	0	0.0	0	0.0	0	0.0	0	0.0	−315	−90.0	0	0.0
NesQuick cereal	Nestle	0	0.0	−0.2	−5.0	−0.7	−43.8	−1.9	−6.0	10	4.2	3	57.7
Plus fibre lift	Uncle Tobys	−40	−2.7	−1.2	−40.0	−0.5	−62.5	−2.5	−9.6	20	19.0	2.4	17.6
**Canned legumes**													
Baked beanz	Heinz	−150	−28.6	−3.5	−87.5	−0.9	−90.0	−2.1	−31.8	5	1.4	0.7	15.6
**Cheese: hard, cheddar & semi-soft**													
Moo Zoo cheese	Devondale	0	0.0	0	0.0	0	0.0	0	0.0	−300	−36.6		
**Confectionary**													
Wonka red skins	Nestle	0	0.0	0	0.0	0	0.0	0	0.0	84	933.3		
Dairy milk choc furry friends	Cadburys	10	0.5	−0.1	−0.3	−0.5	−2.7	0.7	1.3	72	800.0		
Jurassic bitz and Magic fairy dust	Dollar sweets	40	2.3	4	400.0	0.5	50.0	−5.5	−6.2	2	18.2		
100s & 1000s	Dollar sweets	−60	−3.4	0	0.0	0	0.0	−8.5	−9.6	11	100.0		
Milk choc gold coins	Sorini	−7	−0.3	3.3	12.4	−4.8	−28.6	−10.6	−17.5	27	26.2		
Strawberry clouds	Joo Joos confectionary	50	3.6	0	0.0	0	0.0	−3.7	−5.7	−21	−52.5		
Starburst babies	Mars	−62	−4.5	−0.1	−50.0	0	0.0	−7	−13.5	−26	−53.1		
Allens chicos	Nestle	0	0.0	0.1	10.0	0	0.0	0	0.0	0	0.0		
Allens lollipops	Nestle	20	1.2	−0.7	−70.0	0	0.0	−0.2	−0.3	−2	−6.7		
**Dairy snack foods**													
Calci yum custard	Fonterra	3	0.8	0.1	6.3	0.1	9.1	−0.3	−2.5	2	3.3	0.1	50.0
Yo go mix	National foods	36	5.2	1.6	27.6	0.8	22.2	−0.7	−3.4	−3	−3.6		
Milo mousse	Nestle	36	6.4	2.6	89.7	1.8	78.3	−0.7	−4.3				
**Ice creams** &**milk-based ice confections**													
Paddle pop	Steets	0	0.0	0	0.0	0	0.0	0	0.0	−84	−61.8		
Billabong	Nestle peters	0	0.0	0	0.0	0	0.0	0	0.0	−75	−50.0		
**Jelly**													
Aeroplane jelly	Aeroplane	42	18.8	0	0.0	0	0.0	3.8	35.2	29	966.7		
**Miscellaneous**													
Mainland munchables M&Ms	Mainland	20	1.4	0.4	2.0	0.3	2.4	−3.3	−19.8	18	2.3		
**Milk: flavoured**													
Nesquick	Nestle	−40	−2.3	−3	−100.0	−1.9	−100.0	18.5	22.9	5	100.0		
Milk flavouring straws	Sipahh	28	1.8	0.3	100.0	0.2	200.0	0.3	0.6	6.2	24.1		
**Nut spreads**													
Smooth peanut butter	Kraft	−2	−0.1	−1.1	−2.1	2.1	22.6	0.7	9.5	0	0.0		
**Processed fruit**													
Splat fruit puree	Heinz	−30	−10.7	−0.1	−33.3	−0.1	−100.0	0.4	3.0	8	160.0		
**Processed fruit dried**													
Sultanas mini packs	Sunbeam	−50	−3.7	−0.4	−40.0	−0.7	−70.0	1	1.6	−36	−78.3	−1.3	−21.7
Fruit poles (apple, pear & strawb)	Golden days	90	7.7	1	500.0	0.3	300.0	−7	−11.7	−4	−5.7	−3.6	−24.0
Fruity Bites (strappleberry)	Go natural	−9	−0.6	1.9	23.8	0.9	22.0	5.8	14.6	−2.4	−15.6	2.5	37.3
**Processed meat etc. crumbed or coated**													
Captain birdseye fish fingers	Birdseye	72	9.0	0.2	2.4	0.2	28.6	1		19	8.8		
Batman snacks (now ‘dino snacks’)	Steggles	−18	−2.3	0.3	4.0	−0.5	−25.0	1.1	366.7	−215	−41.0		
Fairy shapes (tempura style chicken	Steggles	−124	−14.4	−2.9	−27.9	−0.7	−25.0	0.8	400.0	−104	−26.1		
**Ready to eat pasta/rice/noodle products**													
Spaghetti shapes	Heinz	20	7.4	−0.1	−25.0	0	0.0	−0.2	−5.4	−175	−50.7	0.2	25.0
Kid’s kitchen	Hormel	0	0.0	0	0.0	−1.6	−45.7	0	0.0	0	0.0		
Spag-a-saurus pasta	SPC	49	17.7	0.2	66.7	0	0.0	−0.9	−19.1	−20	−5.7	0.4	50.0
**Savoury dry snack foods**													
Monster rice sticks	Mammee	70	3.6	−0.5	−2.4	−0.8	−8.6	0.8	10.3	−140	−20.6	−0.7	−6.8
**Snack food bars**													
K time twists	Kelloggs	20	1.4	0.8	13.8	0.4	50.0	0	0.0	−20	−11.1	0	0.0
Milo energy bars	Nestle	−20	−1.3	0.1	1.9	−0.3	−15.0	−7.4	−24.7	0	0.0	−0.7	−8.8
Nutrigrain bar	Kellogs	−10	−0.6	−0.6	−6.1	−0.4	−4.9	0	0.0	0	0.0	−0.2	−10.0
**Soups**													
2 Minute noodles	Maggi	−26	−6.7	2.8	466.7	1.5		−0.5	−50.0	−48	−12.7		
**Yoghurts and drinkable yoghurts**													
Vaalia kids yoghurt vanilla	Parmalat	15	4.0	0.1	3.8	0.2	12.5	0.1	0.9	−23	−31.1		
Fruit flavoured yoghurt fruit mix	Yoplait	−194	−35.3	−0.46	−20.0	−0.3	−20.0	−9	−43.3	−13	−23.2		
HannaMontana yoghurt (CalciYum toy story)	Nestle	−30	−7.0	−0.6	−19.4	−0.5	−22.7	−0.8	−6.1	6	15.8		
Petit miam	Yoplait	−35	−6.8	0	0.0	0.1	3.3	−2	−13.9	−8	−11.1		
Squezzie yoghurt	Dairy farmers	−83	−18.9	0	0.0	0	0.0	−4.3	−26.2	−14	−23.7		
Yoghurt with real fruit Dora explorer	Pauls	−8	−2.2	0	0.0	0.1	6.3	−1.2	−10.8	−21	−28.4		
Smackers fruitilicious yoghurt tube	YoPlait	−92	−20.5	0	0.0	0	0.0	−5.2	−30.1	−23	−33.8		

The total number of products positively reformulated is similar to the total number of negatively reformulated products (Table 2). However, examining only the substantially reformulated products shows that considerably more products were substantially positively reformulated than were substantially negatively reformulated. Some products were simultaneously positively and negatively reformulated and some were simultaneously substantially positively and substantially negatively reformulated (Table 2).

#### Method two: nutrient criteria method

In total, 85 products from the sample were given a classification according to the Healthy Kids Association nutrient criteria in 2009 and 2011. Product categories analysed using this method included sweet biscuits, breakfast cereals, canned legumes, confectionary, nut spreads, plain waters, processed meats, ready to eat pasta/rice/noodles, savoury dry snack foods, snack food bars, and soups. Dairy, jelly, and processed fruit products were excluded from this analysis.

Overall, only five (6%) products improved classification over time. Two products were reclassified from Red to Amber and three from Amber to Green. Reductions in sodium content facilitated positive reformulation in four of these five products (Table 4). No products decreased in healthiness according to this system of classification.

**Table 4:** **Australian child-oriented food products within the sample that improved in classification according to the Healthy Kids Association Nutrient Criteria **[46]**from 2009-2011**

**Product**	**Brand**	**Company**	**2009 Classification**	**2011 Classification**	**Nutrient/s reformulated to enable change in category**
Dino snacks	Steggles	Baiada	Red*	Amber	Decreased sodium
Moo Zoo cheese	Devondale	Murray Goulburn	Red*	Amber	Decreased sodium
Spaghetti shapes	Heinz	Heinz	Amber	Green	Decreased sodium
NesQuick cereal	Nestle	Nestle	Amber	Green	Increased fibre
Cheerios	Uncle Tobys	Nestle	Amber	Green	Decreased sodium

Excludes jelly, dairy and processed fruit products.

*Does not meet Healthy Kids Amber Criteria.

## Discussion

This study sought to monitor the extent and nature of reformulation in 120 child-oriented food products over a 2-year period. Reformulation is viewed as a key emerging strategy to address obesity and chronic disease [14-18] and hence, the reformulation of foods marketed to children may play a role in addressing rising rates of childhood overweight and obesity.

### Is the reformulation meaningful?

Changes to nutrient profiles are unlikely to have a meaningful impact on public health outcomes, such as childhood overweight and chronic disease unless these changes occur on a large scale across products commonly consumed by children. As there is not currently a comprehensive set of reformulation benchmarks or targets to assess whether reformulation of a product is large enough to have a meaningful impact on public health outcomes, two assessment methods were devised during this study. The percentage change method in this study differentiated negligible changes from reformulation by providing a stepwise set of benchmarks to assess and classify the magnitude of reformulation. This method demonstrated that a considerable proportion (35%) of child-oriented food products surveyed were substantially reformulated; a larger proportion than were only moderately reformulated (9%). Using the criteria developed for this study, these results suggest that in many cases reformulation is occurring on a meaningful scale. However, this meaningful reformulation is occurring in both directions - both towards and away from what is considered ‘healthy’.

The present study monitored reformulation over multiple nutrients, revealing that a number of products (15%, n = 18) were positively reformulated for one nutrient, but negatively reformulated for another. This finding has not been previously identified as the majority of existing literature tracks reformulation of single nutrients only (eg. trans fats [31,32] and sodium [37]). Reports of improvements in single nutrients can give an inflated view of reformulation progress, when the reality is more complex.

The present study also clearly differentiates positive from negative reformulation. This methodology differs from many reports on industry reformulation, which focus only on industry commitments or actions towards positive reformulation of products [35,37]. Results clearly demonstrate that since 2009, many nutrients surveyed were reformulated both positively and negatively in similar proportions within the sample. While the proportion of substantially positively reformulated products (15%) is considerably more than the proportion of substantially negatively reformulated products (8%), the total proportion of positively reformulated products (33%) is close to the proportion of negatively reformulated products (26%). Furthermore, the nutrient criteria method, which considered a product’s overall nutrient profile to assess reformulation, found only five products (6%) to be positively reformulated. Collectively, these findings indicate that there is no clear trend of reformulation resulting in healthier products across the sample and suggest that the child-oriented food supply has not greatly improved overall since 2009, despite the current strong political climate encouraging positive reformulation [26,50,51] and promises by food and beverage companies to improve their food marketing practices [52].

Milk products and dishes and cereal products and dishes, are good examples of products where a combination of both positive and negative reformulation was observed. Considering these product categories contribute most significantly to children’s energy intakes [2], these products are appropriate targets for positive reformulation. However, the negative reformulation observed in these categories is equally concerning due to the likely impact on population dietary intakes. The selection criteria used in this study included only products marketed directly to children. Unsurprisingly, the largest product category within the resulting sample is confectionary and cereal bars (33%, n = 39). According to national intake data, these foods contributed approximately only 3-5% of total energy intakes of Australian children aged 4–18 years in 2007 [2]. Further monitoring of reformulation of products highly consumed by children represents a key area of further research to better understand the impact of reformulation occurring within the child-oriented food supply and it’s potential to influence public health outcomes.

This lack of consistent positive reformulation and the combination of positive and negative reformulations occurring within the same product may reflect the commonly reported technological barriers associated with reducing the content of nutrients such as fats, particularly saturated fats, sugars and sodium, due to their contribution to shelf-life, textural and other sensory properties of food [37,53,54]. For example, a decreased fat content is commonly coupled with an increased sugar content to preserve sensory qualities of a product.

### Is reformulation tackling the right nutrients in child-oriented food products?

Childhood overweight and obesity is undoubtedly a serious public health problem for children in the developed world [1,15] and greater access to, and increased consumption of, energy dense foods and beverages are consistently identified as major contributing factors [10,23]. The International Obesity Task Force encourages food companies to market lower energy, more nutritious foods to children as a priority action to address childhood overweight and obesity [15]. These conclusions suggest reformulation of child-oriented food products should focus on reducing energy density, by reducing total fat and sugar content.

Findings from the present study, however, reveal that sodium was the most positively reformulated nutrient (21%) among the sample of child-oriented foods. Despite a third of products surveyed being positively reformulated for at least one nutrient, energy density, total fat and sugars content were positively reformulated in only 5%, 9% and 11% of products respectively.

The focus on sodium reformulation observed in this study reflects the emphasis on sodium reformulation within the literature [24,33,34] and in initiatives such as the Food and Health Dialogue [26], Heart Foundation Tick [24] and UK Food Standards Agency’s salt reduction program [55]. However, these initiatives are aimed at addressing hypertension among adult populations. While sodium reduction in child-oriented food products has positive public health implications as it may help to prevent the development of unhealthy food preferences with obvious public health benefits, hypertension is not common in children [56]. This highlights a need for further debate over which nutrients are the most important in children’s health and a need for policy prioritising the reformulation of these nutrients in child-oriented food products. AWASH has begun to contribute to this discussion in regards to the importance of salt reduction in children’s health [57]. Such policy will enable a more focused approach to reformulation efforts in child-oriented food products.

### Recommendations to regulatory groups: The need for reformulation benchmarks

There is little consensus in the literature on the level of change required to constitute meaningful or acceptable reformulation and this exposes a need to develop standardised reformulation benchmarks. These would facilitate consistent research monitoring reformulation and also allow companies to be recognised for positively reformulating products (eg. through a labeling initiative) and provide a mechanism for preventing tokenistic reformulation. Such labeling initiatives have improved the success of voluntary industry reformulation in many cases in Australia (Heart Foundation’s Tick [24]) and internationally (Swedish Keyhole Symbol and Programme National Nutrition Sante [13]).

In Australia, these benchmarks may be achieved by expanding the current targets in the Food and Health Dialogue to include benchmarks for a wider range of nutrients and food categories including children’s food products. However, the need to meet high or unrealistic reformulation benchmarks in order to make a labeling claim is viewed as a major barrier to reformulation for industry [54,58]. Using a stepwise set of reformulation benchmarks, as used in the present study may increase industry incentive to invest in the development needed facilitate reformulation. This would enable regulatory groups to recognise not only companies making substantial reformulations, but also companies making moderate or gradual changes towards health. Similarly, in order to pose realistic reformulation standards or policy, regulatory groups must consider technological limitations in regard to the levels of nutrients such as sugar and salt required to maintain food safety and shelf-life, as well as levels of fat, sugar and salt to maintain consumer palatability [53,54,59].

In addition to identifying target nutrients for children (eg. sugars in fruit drinks),

food products that contribute highly to children’s overall intake of nutrient of concern (eg. fat/sugar/salt) should be prioritised within reformulation benchmarks and policy. This would focus industry reformulation to ensure reformulations have a meaningful impact upon population dietary intakes and public health outcomes. Finally, the present study reveals many products are being positively reformulated for one nutrient, but negatively reformulated for another. A number of food products have been reformulated as a result of voluntary targets set by the Food and Health Dialogue [26]. However, to avoid misleading consumers, the Food and Health Dialogue should consider assessing overall nutrient profiles, not just single nutrients, to ensure negative reformulation has not also occurred in a product recognised as positively reformulated.

School canteen guidelines are likely to have stimulated some of the positive reformulation occurring in the child-oriented food supply [36], with some products (eg. Mamee Monster Noodle Snacks [60]), actively advertising their eligibility to be sold in school canteens on the packaging. Similarly, the use of product traffic light labeling with g/100g or g/portion thresholds for nutrients may also increase industry incentive to reformulate, as it would enable food companies to make claims about meeting certain nutrient criteria (ie. by obtaining green lights) in addition to encouraging reformulation to avoid red lights eg UK Food Standards Agency Traffic Light Standards [61].

### Methodological Issues

The methods used to assess reformulation in the present study differentiate between negligible changes to nutrition content and reformulation, and suggest a methodology to assess whether reformulation is meaningful. However, these methods are limited by their arbitrary nature and reinforce the need to develop standardised reformulation benchmarks. The nutrient criteria method considers overall nutrition profile rather than single nutrients, however, criteria such as the HKA nutrient criteria set high benchmarks and small reformulations went unrecognised with this method. Furthermore, this method excluded dairy and processed fruit products as insufficient data was collected in 2009 to assess these products against the HKA criteria.

The stepwise benchmarks used in the percentage change provided a more sensitive measure that recognised smaller (moderate) reformulations, however the benchmark used for substantial reformulation (25% change in nutrient content based) is also high. This is supported by the food industry, which expressed that, in most products, achieving a 25% reduction (eg. in saturated fat) in a single reformulation is unrealistic [54]. As a result, this study may not recognise smaller reformulations, which if occurring across a large proportion of products in the child oriented food supply, would likely have a meaningful impact on population dietary intakes and public health outcomes. Furthermore, these benchmarks were also used across all product categories, whereas ideally, benchmarks would vary with product category as in the Food and Health Dialogue [26]. Finally, there are limitations associated with the use of percentage change in nutrient composition as a measure in this method. For example, small absolute changes to nutrient composition may be overrepresented when presented as a percentage change. In view of this, regulatory bodies should consider both percentage and absolute change in nutrient composition across a range of nutrients when setting formalised reformulation benchmarks or targets for policy.

While reformulation is currently topical with the establishment of the Food and Health Dialogue [26], the National Healthy School Canteens Project commencing in 2008 [50], and Blewett’s recent review on labelling [51], the two-year window over which reformulation was studied may have been too brief to recognise some reformulation efforts. For example, Cereal Partners Worldwide report that gradual reformulation of two products surveyed, Nesquick [62] and Milo [63] breakfast cereals, has occurred since 2005 and that Cheerios [64] (also surveyed) was reformulated prior to 2009. Similarly, Kellogg’s has been gradually reformulating sodium content of cereals since 1997 [37].

One potential strategy used to improve nutritional composition of their products is to discontinue products that do not meet agreed nutrient criteria and to launch new products that do. However, as this study only investigated products that were still in circulation in 2011, any changes of this sort were deemed product ‘innovation’ rather than ‘reformulation’ and therefore changes of this sort are unacknowledged in the results.

Finally, as NIP data is updated periodically and methods used to analyze nutrient content vary between manufacturers, the accuracy and currency of NIP data collected at both baseline and follow-up may be questioned. When each product was last analysed for nutrient composition is unknown and likely varied. Consequently, the NIP data collected may not provide accurate representation of products at the time of data collection. For example, if a product underwent changes to nutritional content between 2009 and 2001, but the nutrient content was not re-analysed in this time, this change would not be recognised in the present study. As a result, reformulation occurring between 2009 and 2011 may have been underestimated in this study. Ideally, each product would be independently tested at both baseline and follow up to generate nutrient composition data, however budgetary and time constraints in the present study were prohibitive of this approach.

### Implications for further research

Clearly further research using larger sample sizes is needed to monitor reformulation over a longer period of time in order to capture reformulation occurring on a gradual scale. Monitoring reformulation across a number of nutrients and product categories (including child-oriented food products) will provide an overall perspective of the effect of reformulation on the whole food supply. As methods of reporting and assessing reformulation vary greatly in current literature, it is difficult to compare results of the present study to those previously reported, and also to compare results between existing studies. The use of consistent benchmarks to assess reformulation in future research will enable comparison results between studies. Regularly updating nutrient composition databases with changing NIPs of individual products will also facilitate this research. Modeling the effect of specific reformulations of population dietary intakes in children [34] may also be of benefit in identifying where best to target reformulation initiatives, the level of reformulation to aim for (ie. where to set benchmarks) and which nutrients or food products groups to target.

## Conclusions

This study applied a set of arbitrary criteria to judge the extent to which child-oriented foods in Australia had undergone reformulation. The findings of the study highlight three important issues concerning reformation of child-oriented foods in Australia. Firstly it suggests that reformulation of nutrients is not trending towards improving the healthiness of child-oriented food products and that there has been little overall improvement in the Australian child-oriented food supply since 2009. Whilst a considerable proportion of Australian child-oriented food products in this study were substantially reformulated, this reformulation moved products both towards and away from what is considered healthy. Secondly, the study highlights the need for standardised reformulation benchmarks and to identify priority nutrients and product categories to reformulate in order to guide industry reformulation efforts and effectively improve population dietary intakes among children. The setting of standards also raises the possibility of on-going regular monitoring of the food supply to assess the extent to which these standards are being met. Finally, further research monitoring reformulation of large samples of Australian food products across multiple nutrients over a longer time period is required to order to assess if reformulation is an effective strategy in addressing chronic disease, including childhood obesity. This study provides an important starting point for on-going dialogue between food and health industry professionals on the need for agreed standards and methodologies for reformulating child-oriented foods.

## Competing interests

The authors declare that they have no competing interests.

## Authors’ contributions

JC, KM and TU conceptualised the research, participated in the research design and reviewed and commented on all drafts. SS participated in the research design, undertook the data collection and fieldwork in 2011, undertook the analysis and presentation of data, and wrote all drafts including the final manuscript. All authors read and approved of the final manuscript.

## Pre-publication history

The pre-publication history for this paper can be accessed here:

http://www.biomedcentral.com/1471-2458/13/836/prepub
